# Supplementary material for: Digital holography-based 3D particle localization for single-molecule tweezer techniques
Source: Biophys J. 2022 Jun 3;121(13):2538–49. doi: 10.1016/j.bpj.2022.06.001 (PMC9300656; doi:10.1016/j.bpj.2022.06.001)
Supplement: Document S2. Article plus supporting material [file mmc9.pdf]

Biophysical Journal
Article

Biophysical Society

Digital holography-based 3D particle localization for single-molecule tweezer techniques

James L. Flewellen,[1,2] Sophie Minoughan,[1] Isabel Llorente Garcia,[3] and Pavel Tolar[1,2,*]
[1]Immune Receptor Activation Laboratory, The Francis Crick Institute, London, United Kingdom; [2]Institute of Immunity and Transplantation, Division of Infection and Immunity, University College London, London, United Kingdom; and [3]Department of Physics and Astronomy, University College London, London, United Kingdom

ABSTRACT We present a three-dimensional (3D) imaging technique for the fast tracking of microscopic objects in a fluid environment. Our technique couples digital holographic microscopy with three-dimensional localization via parabolic masking. Compared with existing approaches, our method reconstructs 3D volumes from single-plane images, which greatly simplifies image acquisition, reduces the demand on microscope hardware, and facilitates tracking higher densities of microscopic particles while maintaining similar levels of precision. We demonstrate utility of this method in magnetic tweezer experiments, opening their use to multiplexed single-molecule force spectroscopy assays, which were previously limited by particle crowding and fast dissociation times. We propose that our technique will also be useful in other applications that involve the tracking of microscopic objects in three dimensions, such as studies of microorganism motility and 3D flow characterization of microfluidic devices.

SIGNIFICANCE Tracking objects in three dimensions is a common task in biology but typically requires the acquisition of image stacks, which is limited by speed, the depth of field of microscope objectives, and the presence of other objects that obscure the illumination. Here, we develop HoloMiP (holographic microscopy with parabolic masking), which uses digital holography to reconstruct the three-dimensional images from a single plane, allowing tracking of light-scattering objects in three dimensions. HoloMiP outperforms existing methods in precision, speed, simplicity, and tolerance to crowding. We show that it is particularly suitable for fast, multiplexed magnetic tweezer experiments, opening new avenues to high-throughput force spectroscopy.

INTRODUCTION

Tracking microscopic objects in a fluid environment is common in biology. It is used in magnetic and optical tweezers (1), in microfluidic devices (2), for quantifying fluid flows around cells (3), and for direct analysis of the motion of particles, microorganisms, or cells (4). While two-dimensional (2D) tracking in the image (*xy*) plane is well established, many applications call for information along the third dimension, parallel to the optical axis (*z*). 3D tracking is challenging because capturing the motion of objects through the imaged volume typically requires acquisition of *z*-resolved image stacks, which severely limits time resolution. For single light-scattering objects, continuous 3D acquisition is facilitated by the use of look-up table techniques, which infer the *z* position by matching interference rings around the object to reference image stacks (5). However, such techniques require access to each tracked particle prior to or post-tracking to acquire reference z stacks and are limited by the depth of field of microscope objectives and by the presence of other objects in the sample, which obscure the illumination source.

Magnetic tweezers (MT) are a typical imaging application that rely on 3D tracking (6). MTs use an external magnetic field to apply a force or torque to microscopic superparamagnetic beads, which are conjugated to molecules of interest. The position of these microbeads is tracked through time in order to monitor the effects of the external force. This technique has traditionally been used to study force- or torsion-dependent molecular processes, for instance, the extension and torsion of DNA (7,8), the action of helicases and other DNA-binding proteins (9), protein unfolding (10), and force-dependent proteolysis (11). However, the technique also shows promise in probing the kinetics of single-molecule ligand binding at low applied forces and with high throughput (6,12–14). The full potential of MTs in

Submitted November 1, 2021, and accepted for publication May 31, 2022.
*Correspondence: p.tolar@ucl.ac.uk
Editor: Ryota Iino.
https://doi.org/10.1016/j.bpj.2022.06.001
© 2022 Biophysical Society.
This is an open access article under the CC BY license (http://creativecommons.org/licenses/by/4.0/).

this application, however, has not yet been realized due to difficulties arising from the uncertainty about the number of interacting molecules, the non-specific interactions of the microbeads with the imaging surface (15), and also the challenge of tracking the position of the microbeads in three dimensions with sufficient time resolution.

Standard MT protocols localize magnetic microbeads along z by taking advantage of diffraction rings emanating from defocused beads when a coherent illumination source is used. Because the pattern of the diffraction rings depends on the microbead distance from the objective focal plane, the z position of the microbead can be determined by comparing its diffraction pattern with that of a previously determined look-up table (5). The look-up table is a z stack of microbead images recorded by capturing the diffraction rings at known z positions, typically using a nano-positioning stage, while an external magnetic field is applied to lift the microbeads from the surface of the imaging chamber. However, the need to acquire a look-up table for every microbead that needs to be localized severely limits this technique in cases when microbeads move or dissociate during the experiment due to the applied external magnetic field. The cross-correlation algorithm (5,16), which is used to compare the ring pattern with the look-up table, also depends on symmetry and fails when diffraction patterns overlap. This restricts the density of objects that can be tracked in a single field of view. Finally, not all microscopes have a stage with sufficient nano-positioning precision for this technique.

Our technique uses inline digital holographic microscopy (17) to generate a 3D reconstruction of the light field from a single-plane image. Objects are localized in this field as peaks of intensity using a 3D parabolic masking technique. We term our technique HoloMiP (holographic microscopy with parabolic masking). HoloMiP does not require generation of look-up tables nor any a priori knowledge of the objects being imaged and thus simplifies and speeds up imaging. HoloMiP can also localize particles even if their diffraction patterns overlap, improving localization in experiments with high particle density. In addition, its application is not reliant on the symmetry of the imaged objects and could be readily applied to non-spherical microscopic objects that scatter light, such as microflakes (18) or rod-shaped bacteria (19). We demonstrate the utility of HoloMiP in fast, multiplexed single-molecule force spectroscopy assays using MT to apply force at receptor-ligand bonds attached to a surface by DNA tethers.

MATERIALS AND METHODS

MT apparatus

We used an imaging system (Cairn Research, Faversham, UK) based on an inverted scientific microscope (Ti-E Eclipse; Nikon Instruments, Tokyo, Japan) and fitted with oil immersion objectives (CFI60 Plan Fluor 40x, N.A. 1.30 and CFI Plan Apo TIRF, 100x Oil N.A. 1.49) and automated three-axis stage controller (Applied Scientific Instrumentation, Eugene, OR, USA). Semi-coherent illumination was provided by a light-emitting diode ($\lambda = 625$ nm; Thorlabs, Ely, UK) with a single lens. This configuration allowed a sufficiently large working distance between the illumination source and the sample for the MT rig to operate. A pair of 5-mm cube neodymium-iron-boron magnets (Supermagnete W-05-N50-G; Webcraft, Gottmadingen, Germany) was attached to a custom-built bracket that suspended the magnets above the sample but below the light-emitting diode. The illumination passed between the magnets to the sample. These magnets produced a magnetic field gradient in the sample, and the distance between the magnets was able to be adjusted to modify the strength of the magnetic field gradient. The vertical position of the magnets was adjusted by a voice coil actuator and controller (V-277.630 and C-413.2GA, Physik Instrumente, Auburn, MA, USA). The voice-coil actuator could displace the magnets over the full range of motion of 15 mm in about 100 ms. Digital images were recorded by a sCMOS camera (Orca Flash 4.0 v.2; Hamamatsu Photonics, Shizuoka, Japan) and passed to a computer for post-processing. Image acquisition was facilitated by MetaMorph software (Molecular Devices, Silicon Valley, CA, USA).

Flow cells

Flow cells for use in the MT apparatus were made by sandwiching double-sided tape between two microscope coverslips. The floor surface of the flow cell was cleaned by sonication in solutions of ethanol (95%) and 1 M potassium hydroxide. Silica microbeads (3 μm diameter; Bangs Laboratories, Fishers, IN, USA) were partially melted onto the surface to create reference beads, which could be tracked to monitor and correct stage drift. The glass was then treated with silane (2% v/v solution of 3-aminopropyltriethoxysilane in acetone), which was then cured at 110°C for 1 h. This coverslip was then functionalized with a layer of biotinylated polyethylene glycol (MPEG-SVA and Biotin-PEG-SVA; mol wt = 5000; Laysan Bio, Arab, AL, USA). This functionalization allowed us to attach DNA tethers to the floor of the chamber via biotin-streptavidin bonds.

DNA tethers

A tether of length 3.4 kbp (1.15 μm) was made from a single piece of DNA prepared by polymerase chain reaction from a 9.8-kbp pCerOriD plasmid (gift from J. Molloy, Francis Crick Institute, London, UK). One primer contained four biotin molecules, which anchored the tether to the floor of the flow cell via biotin-streptavidin bonds. The other primer had functional groups for attachment to the antigen of interest (-thiol, -Digoxygenin). Both were from Integrated DNA Technologies (Coralville, IA, USA).

Conjugation of CR2 protein to DNA tethers

For dissociation measurements, CR2 protein (Bio-Techne, Minneapolis, MN, USA) was conjugated to the thiol-modified DNA tethers using sulfo-SMCC (Pierce, Appleton, WI, USA). Briefly, the tethers were buffer-exchanged into conjugating buffer (PBS [pH 7.4], 1 mM EDTA) using desalting columns (Pierce) and incubated with 1 M DTT for 30 min at room temperature. CR2 was exchanged into conjugation buffer and incubated with 2.5 mM of Sulfo-SMCC (Pierce), with agitation for 30 min at room temperature. The tether and CR2 were then buffer exchanged into fresh conjugation buffer, mixed, and incubated for 1 h at room temperature. Finally, the sample was buffer exchanged into PBS.

Antibodies and conjugation to magnetic beads

Antibodies specific for CR2 (clone b-ly4, BD Biosciences, Franklin Lakes, NJ, USA) or for digoxigenin (sheep anti-digoxigenin, Bio-Rad Laboratories, Hercules, CA, USA) were conjugated to 2.8 μm-diameter

superparamagnetic Dynabeads (M270 Epoxy; Thermo Fisher Scientific, Waltham, MA, USA) using the manufacturer's protocol. Briefly, 5 mg of beads were resuspended in 1 mL of 0.1 M sodium phosphate buffer pH 7.4, incubated with tilt rotation for 10 min, and washed once in this buffer. One-hundred μg of the antibody was buffer exchanged into 0.1 M sodium phosphate buffer (pH 7.4). Equal volumes of the beads, the antibody, and 3 M ammonium sulfate in 0.1 M sodium phosphate buffer (pH 7.4) were incubated together for 16–24 h at 37°C with tilt rotation. The beads were then washed twice in PBS containing 0.1% (w/v) BSA.

Force calibration

In our MT apparatus, the force applied to superparamagnetic beads in the sample chamber is a function of magnet height above the sample. This relationship can be deduced by observing the Brownian motion of DNA-tethered beads under the influence of the magnetic field gradient and applying the expression $F_{applied} = \frac{k_B T \langle z \rangle}{\langle \delta x^2 \rangle}$, where k_B is the Boltzmann constant, T the absolute temperature, $<z>$ is the extension of the DNA tether along the optical axis, and $<\delta x^2>$ is the variance of the bead position (20).

To calibrate the MT system, we used a 48-kbp length of λ-DNA with biotin molecules incorporated into one end and digoxigenin into the other. The DNA tether attached at one end to the flow cell floor via biotin-streptavidin bonds and at the other to a Dynabead coated in anti-digoxigenin antibodies. We found our system can apply forces of up to 15.9 pN.

HoloMiP

The bespoke image processing and object localization routines of HoloMiP were written in Matlab (R2018a; The MathWorks, Portola Valley, CA, USA). The HoloMiP routines can be sent to multiple CPU cores for parallel processing for shorter reconstruction times. The software is available at https://github.com/jflew/HoloMiP.

HoloMiP first requires relevant physical parameters for the holographic reconstruction as input, namely, wavelength of illumination, refractive index of medium, effective pixel spacing, and z range and resolution for reconstruction.

Additional processing parameters optimize 3D localization of objects in the holographic reconstruction: the intensity threshold over which an initial candidate is identified in the image plane, an option for a Gaussian low-pass filter of the image to reduce high-frequency noise, and the dimensions of the cuboids deployed around each initial guess for the x,y,z position of an object candidate, inside which the parabolic masking subroutine runs.

HoloMiP then performs a back propagation of the illumination field using the Rayleigh-Sommerfeld formalism (17,21) at each specified z position to result in a 3D volume of electromagnetic field intensities (Fig. 1 *b* and *c*).

A hologram ($I_H(x,y,0)$) results from the interference of an object term, $\tilde{O}(x,y,0)$, which arises from light scattered off the sample, with an unscattered reference term, $\tilde{R}(x,y,0)$:

$$I_H(x,y,0) = |\tilde{R}(x,y,0) + \tilde{O}(x,y,0)|^2,$$

where $z = 0$ is denoted the hologram plane (focal plane).

For our purposes, a separate recording of a background image can be used as a good approximation of the intensity of the reference term: $|\tilde{R}(x,y,0)|^2$

The holographic reconstruction procedure requires the object term first to be isolated, which we achieve through normalizing the recorded hologram using the background image:

$$\tilde{O}(x,y,0) \cong \frac{I_H(x,y,0) - |\tilde{R}(x,y,0)|^2}{2\sqrt{|\tilde{R}(x,y,0)|^2}}.$$

To reconstruct the electromagnetic field $\tilde{O}'(x,y,z)$ at a given position, z, along the optical axis, we convolve the recorded hologram object term $\tilde{O}(x,y,0)$ with the Rayleigh-Sommerfeld propagator:

$$\tilde{O}'(x,y,z) = \tilde{O}(x,y,0) * \widehat{H}(x,y,z),$$

where $\widehat{H}(x,y,z) = -\frac{1}{2\pi}\frac{\partial}{\partial z}\frac{e^{ik\rho}}{\rho}$.

$\rho = \sqrt{[(x_0 - x)^2 + (y_0 - y)^2 + (0 - z)^2]}$, the distance between a given coordinate in the hologram (x_0,y_0,0) and a reconstructed position (x,y,z). $k = -\frac{2\pi n}{\lambda}$, the wavenumber of light with wavelength λ and in a sample medium of refractive index n.

HoloMiP performs this operation in Fourier space, using 2D fast Fourier transforms:

$$\tilde{O}'(x,y,z) = \mathcal{F}^{-1}\{\mathcal{F}\{\tilde{O}(x,y,0)\}\mathcal{F}\{\widehat{H}(x,y)\}\}$$

The use of the Fourier transform allows for a low-pass Gaussian filter to be applied to the recorded hologram, if desired, to smooth high-spatial frequency noise, with minimal additional computational overhead.

The procedure is repeated for each user-defined z value, resulting in a 3D electromagnetic field representing the recorded sample. The square of this field is then taken to provide an 3D intensity map (Fig. 1 *c*).

Object localization

The next phase of the HoloMiP algorithm is to localize in 3D space the objects in the sample. Adapting the approach taken in (22), we find identifying features in the gradient of the intensity field more reliable than working on the intensity field itself. We compute the gradient along the z direction by applying a Sobel-like kernel:

$$S = \begin{matrix}[S1]\\ [S2]\\ [S3]\end{matrix}$$

$$S1 = \begin{bmatrix} 1 & 2 & 1\\ 2 & 4 & 2\\ 1 & 2 & 1\end{bmatrix},\ S2 = \begin{bmatrix} 0 & 0 & 0\\ 0 & 0 & 0\\ 0 & 0 & 0\end{bmatrix},$$

$$S3 = \begin{bmatrix} -1 & -2 & -1\\ -2 & -4 & -2\\ -1 & -2 & -1\end{bmatrix}.$$

Fig. S1 demonstrates the effect of applying this gradient kernel to the 3D intensity field, and Fig. S2 demonstrates the effect the kernel application has on microbead localization along the optical axis.

Application of the kernel is computationally expensive. Thus, to minimize the volume over which the kernel is applied, we first localize the objects of interest in the hologram plane and extract columnar cuboids (typically 15 pixels across) centered on these values (Fig. 1 *e*). The initial x, y localization uses a 2D peak-finding algorithm on a maximum-pixel projection of the reconstruction volume (Fig. 1 *d*). At this stage, object candidates can be excluded based on their proximity to other objects or the edge of the hologram, if desired.

After application of the Sobel-like kernel (Fig. 1 *f*), an approximate z value for an object position within the columnar sub-volume is determined by a 1D peak-finding algorithm applied to the central line of pixels along the z axis (Fig. S2).

A cuboid sub-volume, typically 15 × 15 × 25 pixels (Fig. 1 *g*) centered on the approximate x, y, z values determined as described above, is then

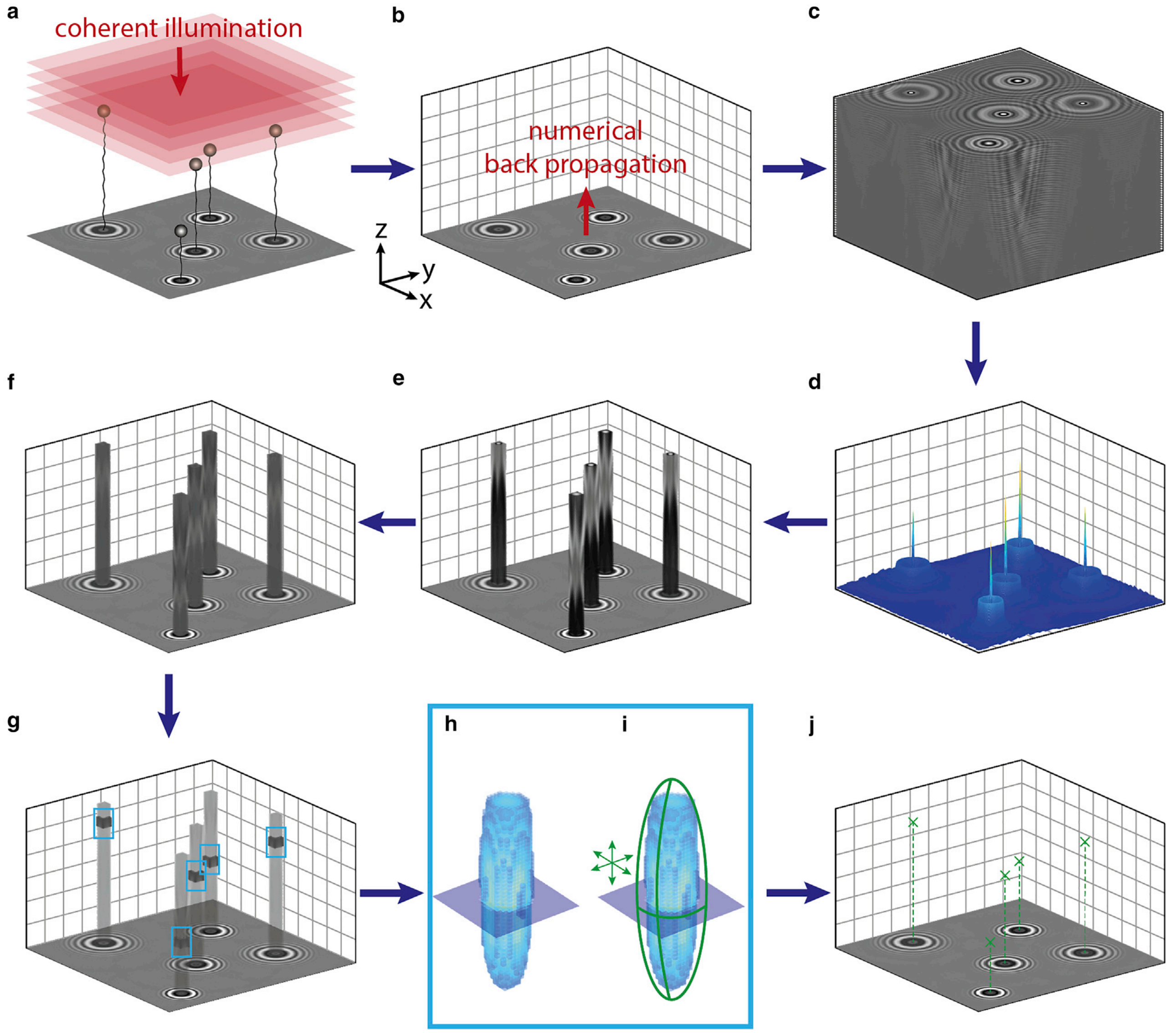

FIGURE 1 HoloMiP algorithm for localizing microscopic objects in 3D. (*a*) Sample is illuminated by a coherent light source, resulting in a recorded digital hologram. (*b*) Rayleigh-Sommerfeld back propagation reconstructs the electromagnetic field at each user-defined plane, resulting in (*c*) a three-dimensional (3D) electromagnetic field over the sample volume. (*d*) An initial guess for the x, y position of each object is found by taking a maximum projection through the intensity of the electromagnetic field, followed by a 2D peak-finding algorithm. (*e*) Columnar cuboids along z and centered on each initial x, y position are extracted. (*f*) A 3D Sobel-like gradient filter is applied to these cuboids. An initial guess for the z position is found as the maximum intensity along z. (*g*) Cuboids around the initial x, y, z guesses are extracted and used for precise localization by parabolic masking. (*h*) shows an isosurface representation of one of the objects. (*i*) A 3D parabolic surface is moved around the cuboid until it matches the data. This results in a precise 3D position for the objects (*j*), which can be used for further analysis.

passed to a subroutine for parabolic masking (Fig. 1 *h* and *i*). The voxel intensity values within this cuboid are modeled by a paraboloid, which we use as a proxy for object position (23). Thus, to localize precisely the center of this paraboloid, we did the following:

1. Multiply the sub-volume by a 3D paraboloid of identical size and determine the center of this product by summing voxel intensities along each dimension
2. Determine the difference between the initial and new center positions
3. Repeat this process until this difference converges to a user-defined threshold

The precise 3D positions of all objects in all frames are then stored for subsequent analysis (Fig. 1 *j*). For instance, the 3D position of each object can be tracked through time and correlated to the introduction of the magnetic field gradient from the MT. In this way dissociation events can be observed and dissociation times measured. The extension length of each tethered microbead can be computed also to ensure complete tether extension, from which single-molecule interactions can be inferred.

Computer hardware and processing speeds

For object 3D localization with HoloMiP, we used an Asus workstation (AsusTek Computer, Taipei, Taiwan) with an Intel i9 CPU with 14 cores (i9-7940X CPU; 3.10 GHz; Intel, Santa Clara, CA, USA) and with 128 GB total RAM running Microsoft Windows 10 Enterprise 2016.

Holographic reconstruction is potentially computationally expensive given the iterative nature of applying the propagator to the hologram to build up the 3D volume. In addition, the requirement to hold the reconstructed 3D volume in memory to perform object localization sets conditions on computer RAM.

To minimize computation time, we employed the multiple CPU cores, such that up to 14 frames (of size 1048 px × 1048 px × 100 z slices) can be reconstructed in parallel using Matlab's parfor command. To minimize the burden on computer RAM, we designed HoloMiP to perform localization operations on small sub-volumes around each identified object candidate (Fig. 1 *e–i*).

Compared with single-core computing, our strategy of parallel reconstruction is slower for some of HoloMiP's operations, owing to the additional time taken to transfer data onto different cores. However, this is only apparent for processing of very small numbers (<20) of frames. Over 200 frames, operating HoloMiP on parallel cores results in at least a fourfold reduction in processing time compared with a single-CPU operation.

It is important to note that the 3D localization of objects via parabolic masking is the least computationally expensive part of the routine (taking 500–1000 times less time than 3D reconstruction of the volume from the hologram, for instance). Thus, for parallel core operation, the total computational time of deploying HoloMiP is almost independent of the number of objects in the field of view. This is not the case in most other comparable 3D object-localization implementations, where each object must be localized sequentially in each frame in a dataset.

On our workstation, a hologram of size 1048 × 1048 px and reconstructed to 100 z slices will return 3D positions of all objects in around 1.1 s.

We note that significant reductions in processing times could be made by

- Implementing HoloMiP in a performance-oriented language,
- Using graphics processing units, which are better suited to iterative computation, to perform holographic reconstruction, or
- Implementing HoloMiP on high-performance computer clusters.

Synthetic data

We generated synthetic diffraction patterns simulating microbeads in silico to compare the performance of HoloMiP and the LUT cross-correlation technique as follows.

A tethered microbead was held above the sample surface by applying the MTs. The microscope stage was moved along the optical axis and a z stack of images with a 50-nm spacing was captured. This image stack was converted into a LUT consisting of a stack of radial diffraction profiles as a function of bead distance from focus, using the techniques described in (16).

To generate a synthetic bead diffraction image, a single profile was selected from this LUT, revolved on a polar grid, and then placed on a larger pixel grid (1024 × 1024 px) at the specified location. Multiple diffraction patterns can be generated from different radial profiles and placed where desired. Setting the intensity of the diffraction patterns to be centered on zero ensured overlapping patterns summed appropriately. Poisson-distributed noise was added to simulate camera shot noise. The intensity of the image was rescaled to the range of the pixel values recorded by our microscope camera. The resulting image, or set of images, was then sent to HoloMiP or LUT cross-correlation algorithms for 3D localization.

RESULTS

HoloMiP algorithm

To develop HoloMiP for 3D particle tracking, we used an inverted microscope coupled with MTs similarly as described (24). The sample containing superparamagnetic microbeads tethered to the coverslip is illuminated by a semi-coherent light-emitting diode and imaged with a high-magnification objective focused above the sample. In order to control the vertical position of the tethered beads, a pair of permanent magnets move vertically above the sample using a fast voice-coil actuator.

The semi-coherent illumination produces diffraction patterns of the microbeads on the *xy* image plane (Fig. 1 *a*), which are treated as holograms to recover the 3D positions of the beads (25). We use the Rayleigh-Sommerfeld formalism (26) to reconstruct the 3D electromagnetic field of the illuminated field of view, in a step termed "numerical back-propagation" (21) (Fig. 1 *b* and *c*; Fig. S1 *a–c*; Videos S1, S2, S3, S4, S5, and S6; see materials and methods for the full description of the procedure). Volumes of high electromagnetic intensity are used as proxies for the locations of the microbeads. Subsequent computational steps interrogate the reconstructed 3D electromagnetic field to localize precisely the positions of peaks in the high-intensity volumes. Approximate transverse (x, y) coordinates of each microscopic object are found by first projecting the maximum-intensity pixel in each transverse plane to a single plane, then deploying a 2D peak-finding algorithm (Fig. 1 *d*). Narrow cuboids along the optical axis centered on these positions are examined (Fig. 1 *e*), and a Sobel-like gradient filter (22) is applied to the field in these cuboids (Fig. 1 *f*; Figs. S1 *d–f* and S2; Videos S4, S5, S6, and S7) to identify an approximate z position of each object. A small cuboid around each initial x, y, z object position (Fig. 1 *g*) is then used for precise 3D localization using a 3D version of parabolic masking (23) (Fig. 1 *h–j*). Detailed information on the localization approach can be found in materials and methods.

The reconstructed z position is corrected to account for the mismatch in refractive indices between the immersion oil and the sample medium, which leads to an underestimation of the reconstructed z displacement of an object (27), as well as the systematic z-positioning errors the Rayleigh-Sommerfeld method is known to produce (28). The application of this optical correction results in the retrieval of accurate absolute particle positions. While a correction factor of the ratio between refractive indices based on the small-angle approximation is commonly used (20), we take inspiration from another approach (16) and develop an empirical correction factor. We used a nano-positioning piezo stage to scan immobile microbeads along the optical axis and reconstruct their positions using HoloMiP. We found that it is possible to apply a linear correction to reconstructed z positions ranging between 7 and 12 μm from the focal plane for a 40× objective to recover the absolute positions. Outside of this range, a non-linear correction factor can be applied (see supporting material and Fig. S3).

To characterize the potential computational burden of HoloMiP, we measured processing times on a dedicated

workstation with an Intel i9 CPU with 14 cores and with 128 GB total RAM. With this setup, a typical hologram frame 1024 × 1024 px and reconstructed to 100 z slices takes ~1.1 s to output the 3D positions of all objects. The computational demands are manageable in typical experiments. Furthermore, this time is virtually independent of the number of objects to be localized in the field of view. By contrast, the computational time of the look-up table (LUT) method is heavily dependent on the number of objects in the field of view. Thus, while HoloMiP could be more demanding of computational power than the LUT technique for low densities of objects, at higher densities it shows notable benefits. See materials and methods for more details.

Comparison between HoloMiP and cross-correlation LUT techniques for 3D localization of microbeads

We compared the performance of HoloMiP with the cross-correlation LUT method used commonly in MT experiments (16). To compare x, y localization, a synthetic diffraction profile of a defocused microbead was generated in silico and moved in plane in discrete steps over a background image (Fig. 2 *a–c*). The size of the steps was adjusted from 0.3 to 19.5 nm, and the position of the microbead was determined using both HoloMiP and a cross-correlation LUT technique. The resolving performance of the two techniques was compared by calculating the signal-to-noise ratio (SNR) as the step size divided by the mean standard deviation of the position trace throughout the recording (Fig. 2 *d*). HoloMiP had consistently higher SNRs than the LUT technique due to lower mean standard deviation (0.68 nm for HoloMiP versus 1.63 nm for the LUT). Consistently, the noise value approached the signal around a step size of 0.7 nm for HoloMiP, whereas for the LUT technique, the value was around 1.6 nm. This analysis indicates that under matching conditions HoloMiP is capable of detecting smaller lateral displacements with higher SNR compared with the LUT technique.

To test the localization precision along the optical axis (z) along with x and y, we immobilized microbeads on a glass surface and recorded images at 100× magnification with a defocus of 10 or 20 μm from the sample plane. A nano-positioning stage then moved the bead sample along the optical axis in discrete steps of 10, 30, and 50 nm (Fig. 2 *e–g*). Although the localization precision in x and y was lower than in the in silico experiments, the two techniques performed similarly at a 10-μm defocus; however, at a 20-μm defocus, HoloMiP outperformed the LUT method in z precision: at a 10-μm defocus, the mean standard deviation along x, y, and z was 5.2, 7.7, and 8.0 nm, respectively, for the LUT technique and 5.6, 9.9, and 8.6 nm for HoloMiP. At a 20-μm defocus, the corresponding values for HoloMiP were comparable with a 10-μm defocus along all three directions (4.4 nm for x, 8.1 nm for y, 8.7 nm for z); however, for the LUT technique, the values were only comparable along x (4.2 nm) and y (6.0 nm) but were a factor of two greater along z (17.6 nm). This was reflected in differences in SNRs calculated as above (Fig. 2 *h*).

Thus, we conclude that HoloMiP matches the LUT technique in z-position precision and extends the focus range through which objects can be tracked with high precision.

To compare directly the two techniques in an MT experiment, we analyzed the 3D movement of a 2.8 μm-diameter Dynabead, attached to the surface via a DNA tether, in response to an applied magnetic field (Fig. 2 *i–l*). The bead is tethered to a 16.3 μm length of λ-DNA via multiple biotin-streptavidin attachments at the glass surface and digoxigenin-anti-digoxigenin at the bead. At the start of the trace, the DNA tether is fully extended through a force of 15 pN induced by the magnets acting on the bead. The magnets are then removed, and the position of the microbead is tracked as it sediments to the floor of the chamber. The magnets are then reintroduced, and the bead lifts away from the surface, re-extending the tether. The outputs of HoloMiP and the LUT technique were in close agreement, although not identical. We noted a lateral offset between the two techniques, which increases linearly with microbead distance from the focus (Fig. S4) and is independent of bead position in microscope field of view. We hypothesize that HoloMiP is sensitive to the alignment of the optical axis to the camera sensor in a way that the LUT technique is not. The LUT technique involves averaging a 2D diffraction pattern to produce a 1D radial profile to be compared with a LUT. This method imposes a radial symmetry on the diffraction pattern, removing any eccentricity in the diffraction ring patterns. Elliptical ring patterns would occur when the optical axis is not perfectly normal to the imaging plane. This averaging does not occur with the holographic reconstruction in HoloMiP, resulting in a sensitivity to an angle between the normal of the imaging plane and the incident illumination. It is important to note firstly that this effect is small and, secondly, that it does not affect the determination of relative displacement of objects through time, which is used to determine relevant biophysical parameters. If desired, the two approaches can be made to agree using a simple linear correction factor (see supporting material).

HoloMiP is able to localize high densities of particles

A weakness of the cross-correlation LUT technique is a tendency to fail when the diffraction patterns from adjacent objects overlap. To assess how HoloMiP performs in localizing particles with overlapping diffraction patterns, we compared the two techniques in experiments with in silico synthetic diffraction patterns of microbeads. First, we performed a "crowding" experiment, whereby a central microbead was surrounded by up to eight crowding beads in the same xy

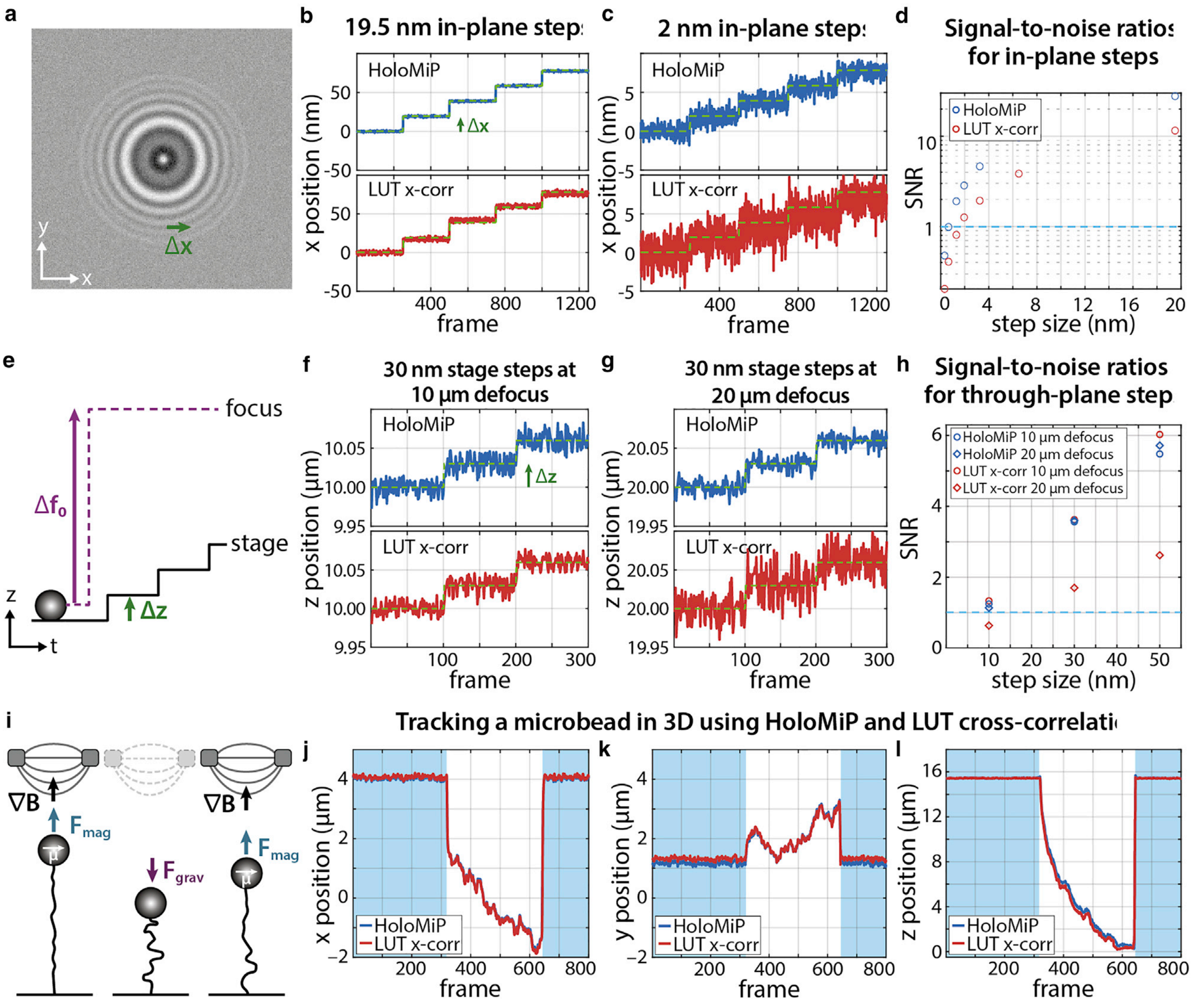

FIGURE 2 Comparison of HoloMiP and look-up table (LUT) cross-correlation for localizing superparamagnetic microbeads. (*a*) An in silico synthetic diffraction pattern, positioned 10 μm from the focus, is moved in plane by discrete steps (Δx). (*b* and *c*) The *x*, *y* localization through time of the diffraction pattern moving in steps of 19.5 nm (*b*) and 2 nm (*c*) are shown for each technique. The mean standard deviation was 0.68 nm for HoloMiP and 1.63 nm for the LUT. (*d*) The signal-to-noise ratios (SNRs) are shown for each technique. The dashed line shows an SNR value of 1. (*e*) The through-plane resolution of the two techniques is tested by using a nano-positioning stage to move an immobilized microbead in steps (Δz) along the optical axis (*z*) after an initial defocusing of the microscope objective (Δf_0). Resolution of 30-nm steps with a 10- (*f*) and 20-μm defocus (*g*). (*h*) The corresponding SNR shows the two techniques are similar at a 10-μm defocus; however, HoloMiP outperforms the LUT technique at the 20-μm defocus. (*i*) The magnetic field gradient ($\nabla\mathbf{B}$) from two permanent magnets induces a magnetic dipole ($\boldsymbol{\mu}$) in, and exerts an upwards force ($\mathbf{F}_{mag}$) on, a 2.8-μm magnetic microbead tethered to a 16.3-μm length of DNA. When the magnets are moved out of position, the microbead descends to the surface under gravity ($\mathbf{F}_{grav}$). (*j–l*) The position of the microbead in response to the magnetic field is tracked through time in 3D using both HoloMiP and the LUT technique; blue shading indicates when a magnetic force of 15 pN is applied.

plane and at distances ranging from 8 to 24 μm, corresponding to ∼3–8 bead diameters (Fig. 3 *a–c*). We recovered the *z* position of the central bead in all conditions as the distance from the focal plane varied from 0 to 30 μm using HoloMiP or the LUT technique and compared them with a baseline position determined from images containing the central bead only (Fig. 3 *d* and *e*). As expected, the LUT technique failed when bead separation approached 10 μm (∼3.6 bead diameters), especially at greater focal distances, where the diffraction rings are larger (Fig. 3 *d* and *e*). By contrast, HoloMiP was able to recover the localization of the central bead in all conditions, up to a 30-μm defocus. As an indication of localization precision, the magnitude of the greatest difference between the baseline bead location and HoloMiP's localization was ∼0.6 μm along *z* (approximately one-fifth of the microbead diameter) and ∼0.04 μm within the image plane. Overall, these results show a significant advantage of HoloMiP with respect to LUT techniques for the localization of closely spaced diffracting microbeads.

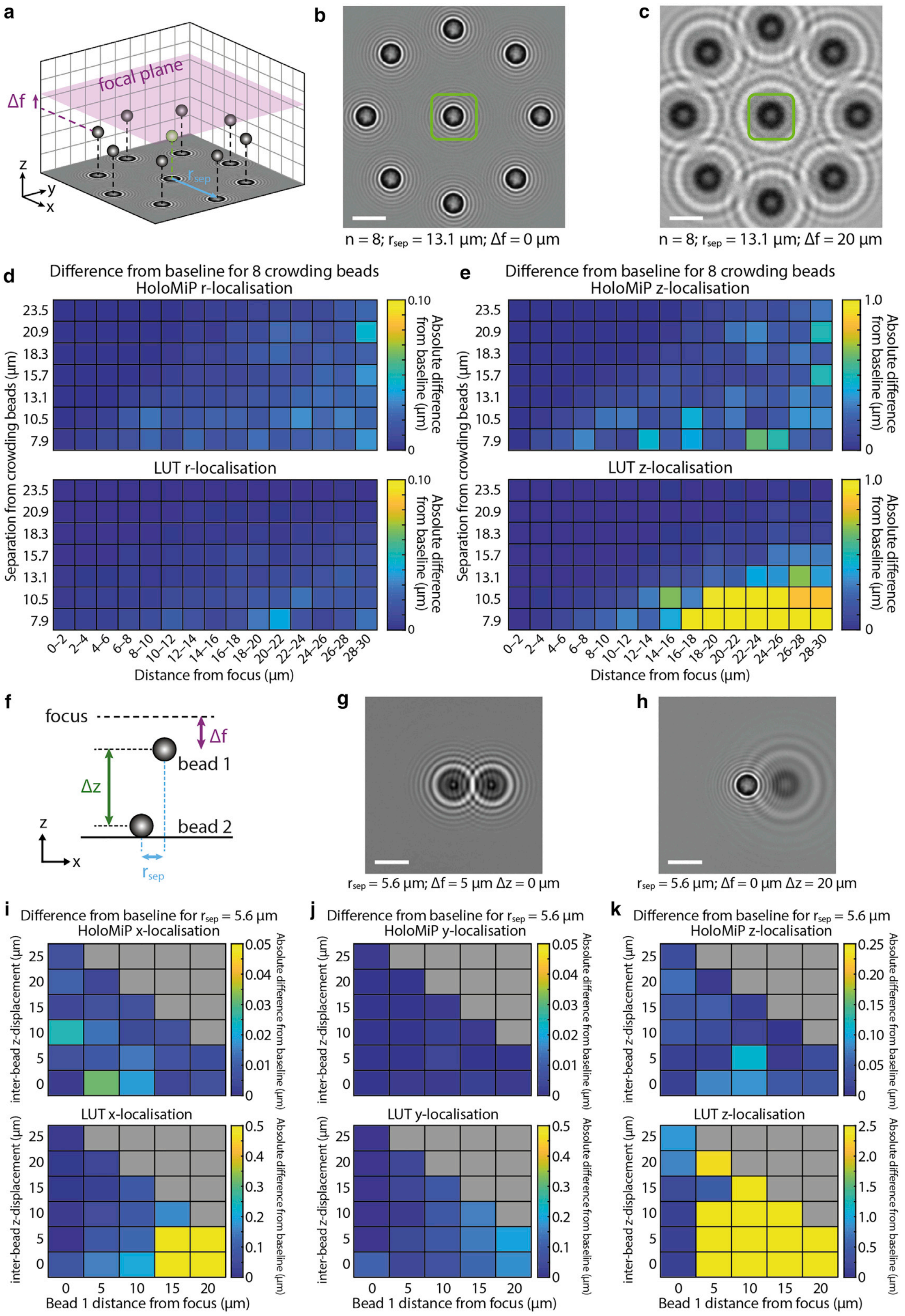

FIGURE 3 HoloMiP is successful at localising microbeads in 3D when their diffraction rings overlap. (*a*) Schematic for an in silico synthetic diffraction pattern bead "crowding" experiment. Up to eight microbeads (n) surround a central bead (in *green*) at a range of separation distances (r_{sep}). The plane containing the microbeads is displaced from the focal plane by Δf, which ranges from 0 to 30 μm. (*b*) and (*c*) show two example fields of view for n = 8. The 3D position of the central bead is recovered using both HoloMiP and the LUT technique and compared with the positions found for a baseline image with no

(legend continued on next page)

Second, we investigated the effect of crowding beads colocalized closely in x and y but displaced along z. In in silico synthetic diffraction-pattern bead experiments with two beads, we varied the position of the central bead (bead 1) from the focus and the z distance of the crowding bead (bead 2) from the first bead (Fig. 3 *f–h*). We also assessed effects of such crowding when the crowding bead was displaced in x by one to two bead diameters, which is a realistic scenario in MT assays. Again, the LUT failed to recover the localization of the bead of interest (Fig. 3 *i–k*), except in some cases where the central bead was sharply in focus, while HoloMiP was able to recover the localization of the central bead in all conditions tested (Fig. 3 *g* and *h*). As an indication of localization precision, the greatest difference between the baseline bead position and HoloMiP's localized position along x was ~0.03 μm when the microbeads were separated by two bead diameters and ~0.05 μm with a single bead-diameter displacement. The corresponding values along z were ~0.11 and ~0.23 μm. Effects along y were negligible.

Measurement of force-mediated dissociation of antibody-antigen bonds using HoloMiP

To demonstrate the advantages of HoloMiP for a new MT application, we designed a system to measure dynamic single-molecule bond dissociation. Using a LUT in such applications limits the number of microbeads that can be analyzed in the field of view and prevents measurement of fast dissociation because of the need to acquire a z stack prior to each measurement. Our experimental system consisted of antibody-conjugated superparamagnetic microbeads, bound to antigen covalently attached to a DNA tether, in turn bound to the surface of the imaging coverslip via multivalent streptavidin-biotin interactions (Fig. 4 *a*). The application of magnetic forces extends the DNA tether before bond dissociation, thus separating the antibody-antigen interaction from the chamber surface. This has two advantages. First, it eliminates binding contributions from non-specific interaction between the antibodies or microbeads with the surface. Second, by measuring the extension length of the DNA tethers before dissociation, beads tethered through multivalent interactions can be eliminated from the kinetic analysis of dissociation as they show shorter tether extension (Fig. S5 *a*).

We applied this technique to the binding of complement receptor 2 (CR2) to anti-CR2 antibodies. The anti-CR2 antibodies were coated onto Dynabeads, and the CR2 was covalently attached to 3.4-kbp DNA tethers, which also incorporated biotin molecules at the opposite end. The CR2 tethers and the anti-CR2 microbeads were pre-incubated and bound to the imaging chamber surface through biotin-streptavidin interactions (Fig. 4 *a*). After 5 s of recording, the desired force was applied by moving the MTs into position. Microbeads would extend their DNA tethers in response to the applied force, followed (in most cases) by a dissociation event, marked by the microbead disappearing from the field of view (Fig. 4 *b*). Data sets lasting for 30 min were recorded for three different applied forces: 3.7, 7.1, and 9.9 pN. A typical data set would have several tens of microbeads for analysis.

HoloMiP was used to reconstruct the 3D positions of each microbead through time. Subsequent analysis determined the height above the sample chamber floor that each microbead reached prior to dissociation and the time of dissociation (Fig. 4 *b*). Our analysis showed that only a subset of microbeads reached full tether extension (1.15 μm) in an immediate response to the applied force (Fig. S5 *c–e*). A number of other microbeads moved to some height lower than full tether extension before dissociating. Some other microbeads moved in z through time in a clear stepwise manner, indicating the presence of multiple tether-bead interactions that were dissociating one by one (Fig. S5 *b*).

To investigate the dependence of the CR2-anti-CR2 dissociation on mechanical load, we considered only microbeads that responded immediately to the applied force and that extended cleanly to at least 1.0 μm (87% of the theoretical full tether extension). This value was chosen as a compromise between the likelihood of a single-molecule interaction and sufficient numbers of microbeads for analysis. Bead dissociation through time was plotted for each applied force, and bond lifetimes derived from the fit of single-phase exponential decays to the data (Fig. 4 *c*). The bond half-life values (t_F) as a function of applied force (F) can then be calculated, yielding data that fit Bell's equation for slip bonds (29):

$$t_F = t_0 exp\frac{-Fx_b}{k_B T},$$

where t_0 is the zero-force bond half-life, x_b is the reaction coordinate, k_B is Boltzmann's constant, and T is absolute temperature (Fig. 4 *d*). The results of this analysis indicated a t_0 value of 149 s and x_b of 0.58 nm. These values are in the range of the expected half-lives of antibody-antigen bonds and agree with the mechanical strength of similar

crowding. (*d*) and (*e*) show variation from this baseline for the mean of 2-μm intervals of ΔF for both in-plane ($r = \sqrt{x^2 + y^2}$) and z localizations. Note that the LUT technique is less successful at z localization at greater focus distances. (*f*) Schematic for a synthetic diffraction pattern bead experiment where a single crowding bead (bead 2) is positioned closely to the central bead (bead 1) along x, but the z displacement (Δz) between the beads is varied (*g*) and (*h*). (*i*), (*j*) and (*k*) show the effect of the crowding bead on the precision of localization of the central bead when compared with a baseline value with no crowding bead. Note that the scale on the LUT plots is an order of magnitude different to that on the HoloMiP plots. Gray squares indicate experimental conditions not tested. Scale bars are 5 μm.

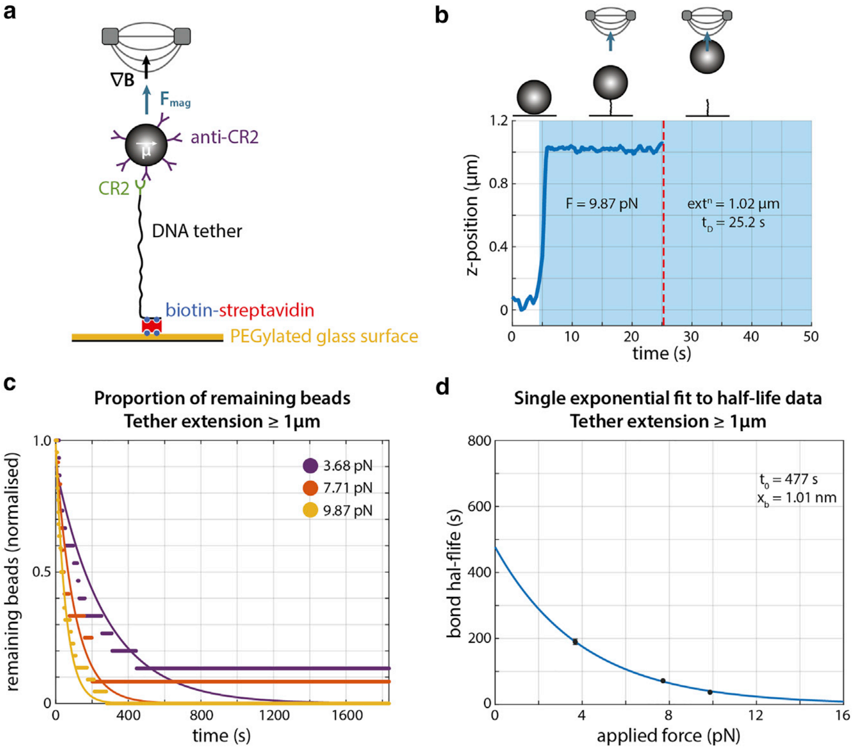

FIGURE 4 Application of HoloMiP to magnetic tweezer assays studying force-mediated single-molecule dissociation. (*a*) Experimental setup to probe dissociation between CR2 and anti-CR2 antibodies. (*b*) Example *z* trace of a tethered microbead subject to a 9.9-pN force. Blue shading indicates when the magnetic field is applied; dissociation time is taken when the microbead disappears from the field of view (*red line*). Images are acquired at 2 fps before the magnetic field is applied, 5 fps for the first 30 seconds after the magnetic field application, and 2 fps for the duration of the recording. (*c*) Proportion of microbeads, which extended to at least 1.0 μm from the chamber surface, remaining attached in response to three different applied forces. Single exponentials have been fitted to determine k_{off} values (F = 3.7 pN, N = 13, $R^2 = 0.986$; F = 7.71 pN, N = 11, $R^2 = 0.986$; F = 9.9 pN, N = 22, $R^2 = 0.989$). (*d*) Corresponding half-life values with single exponential fit to the Bell model (*blue line*). The fit results in t_0 of 149 s and x_b of 0.58 nm ($R^2 = 0.999$).

antibody-antigen interactions measured by atomic force microscopy (30,31) (Fig. 4 *d*).

By contrast, the same analysis could not be performed as well on the subset of beads that only extended to a height less than 1.0 μm. Single-phase exponential decays could only be fitted by including a substantial vertical offset term, to account for the number of microbeads that remained tethered at the end of data acquisition (after 30 min), indicating the presence of multiple tethers or other long-lived non-specific interactions (Fig. S5 *d*). Thus, fast measurement of bead height before dissociation improves the quality of the force spectroscopy data.

DISCUSSION

We have developed a novel 3D imaging technique we term HoloMiP and applied it to perform force-mediated single-molecule dissociation experiments using MTs.

HoloMiP uses a single recorded image to reconstruct the 3D positions of microscopic objects in the sample. Compared with conventional MT techniques, HoloMiP does not require any a priori stage movement nor engagement of the magnetic field. Thus, it is ideally suited to measure fast dissociation events. HoloMiP is also superior to conventional LUT cross-correlation techniques in localizing greater densities of objects in 3D.

Other holographic imaging techniques, based on Lorenz-Mie theory, have been developed (32,33), and although they are typically slower, their speed has been improved by recent advancements (33). The Rayleigh-Sommerfeld formalism we have used is easier and faster to implement but suffers from measurement errors along the optical axis (28,34). We overcome this limitation in HoloMiP through application of the correction factor described in results and with novel gradient filter and parabolic masking techniques, focusing on relative positions of objects to increase accuracy. One further key advantage of the Rayleigh-Sommerfeld implementation, which is retained in HoloMiP, is that it does not require any a priori knowledge of particle size or refractive index.

While the demands on microscope hardware are less onerous for HoloMiP compared with other MT setups, its requirements for computing resources are higher. The holographic reconstruction is computationally intensive; however, this could be mitigated through the use of cluster servers or graphics processing units. The light-emitting diode illumination used limits the effective *z* range of reconstruction through being relatively low in intensity and coherence length. Although not necessary for the application we demonstrate here, laser illumination would overcome these limitations to provide a greater depth of field for 3D tracking applications; the best option would be a laser with a sub-millimeter coherence length, such as that used in (17), to avoid additional interference noise arising from dust on optical elements.

Force is increasingly recognized as an important factor in biological processes (35,36). Measuring the dissociation of receptor-ligand complexes under mechanical stress can

thus reveal hidden characteristics of the bonds that are relevant to biology (37). As an example, we show the slip-bond character of an anti-CR2 antibody binding to CR2 with mechanical strength typical for affinity-matured antibodies (30,31). However, with existing single-molecule techniques, it is difficult and time consuming to acquire enough data for new insights into force-mediated single-molecule interactions. HoloMiP is a new technique that can increase the throughput of single-molecule MT force spectroscopy studies. Taking advantage of MT's virtually uniform magnetic field strength across the microscopic field of view containing many magnetic beads (38), HoloMiP's ability to track many microbeads simultaneously—including those with overlapping diffraction patterns—means single-molecule force assays can be multiplexed for higher throughput than existing techniques. In addition, HoloMiP overcomes previous limitations of non-specific surface interactions obscuring the single-molecule behavior by using DNA tethers to separate the interaction of interest from the surface and through instantaneous measurement of the tether extension under applied force. Furthermore, HoloMiP circumvents a pre-calibration step to acquire a LUT, which already requires application of a force to the system. Even a small force may disrupt weak single-molecule interactions. Thus, with HoloMiP, single-molecule interactions with shorter lifetimes can be investigated. Finally, the localization precision along the z direction for data analyzed with HoloMiP is more consistent over a larger defocus distance compared with the LUT method. This opens up MT experiments to a much greater focal range than those currently employed and relieves the need for hardware autofocusing, which could introduce noise. We also note that this means HoloMiP could be adapted easily to other 3D imaging applications, for instance, quantifying fluid flows in microfluidic devices and tracking the behavior of free-swimming cells, microorganisms, and other objects.

SUPPORTING MATERIAL

Supporting material can be found online at https://doi.org/10.1016/j.bpj.2022.06.001.

AUTHOR CONTRIBUTIONS

J.L.F. designed and implemented HoloMiP, validated it on synthetic diffraction images and MT data, and co-wrote the manuscript. S.M. implemented LUT bead localization and carried out MT calibration and CR2-anti-CR2 dissociation experiments. I.L.G. designed parabolic masking for 3D bead localization and co-wrote the manuscript. P.T. conceived and supervised the research and co-wrote the manuscript.

ACKNOWLEDGMENTS

We thank Justin Molloy, Daniel Burnham, and Hassan Yardimci for providing starting materials for the DNA tethers and for help with the cross-correlation look-up particle localization and Antonio Casal for the help with protein conjugation. This work was supported by the European Research Council (Consolidator Grant 648228) and the Francis Crick Institute, which receives its core funding from Cancer Research UK (FC001185), the UK Medical Research Council (FC001185), and the Wellcome Trust (FC001185). This research was funded in whole, or in part, by the Wellcome Trust (grant number FC001185). For the purpose of Open Access, the author has applied a CC BY public copyright license to any Author Accepted Manuscript version arising from this submission.

DECLARATION OF INTERESTS

The authors declare no competing interests.

REFERENCES

1. Neuman, K. C., and A. Nagy. 2008. Single-molecule force spectroscopy: optical tweezers, magnetic tweezers and atomic force microscopy. *Nat. Methods.* 5:491–505. https://doi.org/10.1038/nmeth.1218.
2. Raffel, M., C. E. Willert, ..., J. Kompenhans. 2018. Particle Image Velocimetry, Third edition. Springer International Publishing AG.
3. Guasto, J. S., K. A. Johnson, and J. P. Gollub. 2010. Oscillatory flows induced by microorganisms swimming in two dimensions. *Phys. Rev. Lett.* 105:168102. https://doi.org/10.1103/physrevlett.105.168102.
4. Meijering, E., O. Dzyubachyk, ..., W. A. van Cappellen. 2009. Tracking in cell and developmental biology. *Semin. Cell Dev. Biol.* 20:894–902. https://doi.org/10.1016/j.semcdb.2009.07.004.
5. Gosse, C., and V. Croquette. 2002. Magnetic tweezers: micromanipulation and force measurement at the molecular level. *Biophys. J.* 82:3314–3329. https://doi.org/10.1016/s0006-3495(02)75672-5.
6. De Vlaminck, I., and C. Dekker. 2012. Recent advances in magnetic tweezers. *Annu. Rev. Biophys.* 41:453–472. https://doi.org/10.1146/annurev-biophys-122311-100544.
7. Bryant, Z., F. C. Oberstrass, and A. Basu. 2012. Recent developments in single-molecule DNA mechanics. *Curr. Opin. Struct. Biol.* 22:304–312. https://doi.org/10.1016/j.sbi.2012.04.007.
8. Lipfert, J., M. Lee, ..., N. H. Dekker. 2014. Magnetic tweezers for the measurement of twist and torque. *JoVE* e51503. https://doi.org/10.3791/51503.
9. Burnham, D. R., B. Nijholt, ..., C. Dekker. 2017. Annealing helicase HARP closes RPA-stabilized DNA bubbles non-processively. *Nucleic Acids Res.* 45:4687–4695. https://doi.org/10.1093/nar/gkx147.
10. Chen, H., H. Fu, ..., J. Yan. 2011. Improved high-force magnetic tweezers for stretching and refolding of proteins and short DNA. *Biophys. J.* 100:517–523. https://doi.org/10.1016/j.bpj.2010.12.3700.
11. Sarkar, R., and V. V. Rybenkov. 2016. A guide to magnetic tweezers and their applications. *Front. Phys.* 4:491. https://doi.org/10.3389/fphy.2016.00048.
12. Danilowicz, C., D. Greenfield, and M. Prentiss. 2005. Dissociation of ligand-receptor complexes using magnetic tweezers. *Anal. Chem.* 77:3023–3028. https://doi.org/10.1021/ac050057+.
13. Noy, A., and R. W. Friddle. 2013. Practical single molecule force spectroscopy: how to determine fundamental thermodynamic parameters of intermolecular bonds with an atomic force microscope. *Methods.* 60:142–150. https://doi.org/10.1016/j.ymeth.2013.03.014.
14. Yao, L., and S. Xu. 2012. Force-induced selective dissociation of noncovalent antibody-antigen bonds. *J. Phys. Chem. B.* 116:9944–9948. https://doi.org/10.1021/jp304335a.
15. Jacob, A., L. J. van IJzendoorn, ..., M. W. Prins. 2012. Quantification of protein-ligand dissociation kinetics in heterogeneous affinity assays. *Anal. Chem.* 84:9287–9294. https://doi.org/10.1021/ac301894k.
16. van Loenhout, M., J. W. Kerssemakers, ..., C. Dekker. 2012. Non-bias-limited tracking of spherical particles, enabling nanometer resolution at

low magnification. *Biophys. J.* 102:2362–2371. https://doi.org/10.1016/j.bpj.2012.03.073.

17. Flewellen, J. L., I. M. Zaid, and R. M. Berry. 2019. A multi-mode digital holographic microscope. *Rev. Sci. Instrum.* 90:023705. https://doi.org/10.1063/1.5066556.

18. Nguyen, J., D. V. Conca, …, I. Llorente Garcia. 2019. Magnetic control of graphitic microparticles in aqueous solutions. *Proc. Natl. Acad. Sci. USA* 116:2425–2434. https://doi.org/10.1073/pnas.1817989116.

19. Hook, A. L., J. L. Flewellen, …, M. R. Alexander. 2019. Simultaneous tracking of Pseudomonas aeruginosa motility in liquid and at the solid-liquid interface reveals differential roles for the flagellar stators. *mSystems.* 4:e00390-19. https://doi.org/10.1128/msystems.00390-19.

20. Yu, Z., D. Dulin, …, N. H. Dekker. 2014. A force calibration standard for magnetic tweezers. *Rev. Sci. Instrum.* 85:123114. https://doi.org/10.1063/1.4904148.

21. Lee, S.-H., and D. G. Grier. 2007. Holographic microscopy of holographically trapped three-dimensional structures. *Opt Express.* 15:1505. https://doi.org/10.1364/oe.15.001505.

22. Wilson, L., and R. Zhang. 2012. 3D localization of weak scatterers in digital holographic microscopy using Rayleigh-Sommerfeld back-propagation. *Opt Express.* 20:16735. https://doi.org/10.1364/oe.20.016735.

23. Llorente-Garcia, I., T. Lenn, …, M. C. Leake. 2014. Single-molecule *in vivo* imaging of bacterial respiratory complexes indicates delocalized oxidative phosphorylation. *Biochim. Biophys. Acta Bioenerg.* 1837:811–824. https://doi.org/10.1016/j.bbabio.2014.01.020.

24. Vilfan, I. D., J. Lipfert, …, N. H. Dekker. 2009. Magnetic tweezers for single-molecule experiments. *In* Handbook of Single-Molecule Biophysics. Springer US, pp. 371–395.

25. Gabor, D. 1948. A new microscopic principle. *Nature.* 161:777–778. https://doi.org/10.1038/161777a0.

26. Goodman, J. W. 2005. Introduction to Fourier Optics, Third edition. Roberts & Company.

27. Hell, S., G. Reiner, …, E. H. K. Stelzer. 1993. Aberrations in confocal fluorescence microscopy induced by mismatches in refractive index. *J. Microsc.* 169:391–405. https://doi.org/10.1111/j.1365-2818.1993.tb03315.x.

28. Cheong, F. C., B. J. Krishnatreya, and D. G. Grier. 2010. Strategies for three-dimensional particle tracking with holographic video microscopy. *Opt Express.* 18:13563. https://doi.org/10.1364/oe.18.013563.

29. Bell, G. I. 1978. Models for the specific adhesion of cells to cells. *Science.* 200:618–627. https://doi.org/10.1126/science.347575.

30. Morfill, J., K. Blank, …, H. E. Gaub. 2007. Affinity-matured recombinant antibody fragments analyzed by single-molecule force spectroscopy. *Biophys. J.* 93:3583–3590. https://doi.org/10.1529/biophysj.107.112532.

31. Katletz, S., C. Stroh, …, P. Hinterdorfer. 2010. Force-induced lysozyme—HyHEL5 antibody dissociation and its analysis by means of a cooperative binding model. *Biophys. J.* 99:323–332. https://doi.org/10.1016/j.bpj.2010.03.060.

32. Lee, S.-H., Y. Roichman, …, D. G. Grier. 2007. Characterizing and tracking single colloidal particles with video holographic microscopy. *Opt. Express.* 15:18275. https://doi.org/10.1364/oe.15.018275.

33. Brouwer, T. B., N. Hermans, and J. van Noort. 2020. Multiplexed nanometric 3D tracking of microbeads using an FFT-phasor algorithm. *Biophys. J.* 118:2245–2257. https://doi.org/10.1016/j.bpj.2020.01.015.

34. Kapfenberger, D., A. Sonn-Segev, and Y. Roichman. 2013. Accurate holographic imaging of colloidal particle pairs by Rayleigh-Sommerfeld reconstruction. *Opt. Express.* 21:12228. https://doi.org/10.1364/oe.21.012228.

35. Tolar, P. 2017. Cytoskeletal control of B cell responses to antigens. *Nat. Rev. Immunol.* 17:621–634. https://doi.org/10.1038/nri.2017.67.

36. Huse, M. 2017. Mechanical forces in the immune system. *Nat. Rev. Immunol.* 17:679–690. https://doi.org/10.1038/nri.2017.74.

37. Tolar, P., and K. M. Spillane. 2014. Force generation in B-cell synapses: mechanisms coupling B-cell receptor binding to antigen internalization and affinity discrimination. *Adv. Immunol.* 123:69–100. https://doi.org/10.1016/B978-0-12-800266-7.00002-9.

38. Ribeck, N., and O. A. Saleh. 2008. Multiplexed single-molecule measurements with magnetic tweezers. *Rev. Sci. Instrum.* 79:094301. https://doi.org/10.1063/1.2981687.

Biophysical Journal, Volume *121*

Supplemental information

Digital holography-based 3D particle localization for single-molecule tweezer techniques

James L. Flewellen, Sophie Minoughan, Isabel Llorente Garcia, and Pavel Tolar

Digital holography-based 3D particle localization for single molecule tweezer techniques

SUPPLEMENTARY MATERIAL:

SUPPLEMENTARY NOTES

SUPPLEMENTARY FIGURES 1-5

SUPPLEMENTARY VIDEO 1-7 LEGENDS

James L. Flewellen[1,2], Sophie Minoughan[1], Isabel Llorente Garcia[3], Pavel Tolar[1,2, *]

[1)] Immune Receptor Activation Laboratory, The Francis Crick Institute, London NW1 1AT, United Kingdom

[2)] Institute of Immunity and Transplantation, Division of Infection and Immunity, University College London, London, NW3 2PP, United Kingdom

[3)] Dept. of Physics and Astronomy, University College London, London, WC1E 6BT, United Kingdom

* Corresponding author: p.tolar@ucl.ac.uk

Optical correction for retrieval of absolute particle positions

To determine the optical correction required for 3D imaging with microscope objectives, and to determine the optimal focal range for experiments, a sample was prepared with Dynabeads and silica reference beads immobilised on the imaging chamber floor. The focus of a 40x objective lens was initially positioned at the floor of the chamber. Using a piezoelectric nano-positioner, the objective lens was moved in 0.5 µm steps away from the floor of the chamber over a range of 12 µm. The z positions of the microbeads were then recovered using HoloMiP and compared to the theoretical position of the objective (Supplementary Fig. 3a). We observed a linear relationship between recovered z position and objective position when the objective was positioned between 7 µm and 12 µm from the chamber floor (Supplementary Fig. 3b).

To validate this result further, and to simulate a typical tether extension experiment, we used the same sample and moved the objective away from its focus at the floor of the sample by 12 µm. The imaging stage was then moved over 5 µm in 100 nm steps, bringing the sample closer to the focus (Supplementary Fig. 3c). The z positions were again determined by HoloMiP and compared to the theoretical sample-to-focus distance. The linear relationship between recovered and theoretical position held for both Dynabeads (Supplementary Fig. 3d) and silica reference microbeads (Supplementary Fig. 3e).

It is important to note that while the absolute position in 3D space of the centre of the microbead is not recovered, its position in 3D *relative* to other beads in the sample, and to itself at different time points in a recording is, through the reconstruction of light scattered off the bead using HoloMiP. The electric field distribution of microspheres under similar imaging conditions is discussed in (1). Operating within the linear focus regime of the objective lens ensures that comparison of relative positions of microbeads is trivial. It is also possible to apply a non-linear correction to samples recorded outside of this range, as long as this relationship has been determined.

Microbead position offset between HoloMiP and LUT approaches

There is a small lateral offset when comparing the recovered positions found using HoloMiP and the conventional look-up table approach. The magnitude of this offset increases linearly with the microbead distance away from the objective focus (Supplementary Fig. 4). We thus posit that HoloMiP is more sensitive to the alignment of the optical axis to the camera sensor. To quantify this offset, we used the two techniques to analyse a force calibration dataset, where a Dynabead is

tethered to a 16.3 µm length of λ-DNA and subject to an increasing magnetic field. This dataset yields 40,000 images of a Dynabead at a range of displacements from the microscope focus. The difference in the output of the two techniques was computed and a linear fit applied along both x and y axes to determine the lateral offset as a function of microbead z position. The difference between the two techniques was found to vary by 17.19 ± 0.03 nm along x and 22.09 ± 0.03 nm along y per 1 µm displacement from the focus (uncertainty is 95% confidence bound on the linear fit). This correction factor can be used to make the two techniques to agree with each other.

Supplementary References

1. Krivitsky, L. A., Wang, J. J., Wang, Z. & Luk'yanchuk, B. Locomotion of microspheres for super-resolution imaging. *Sci Rep* **3,** 165 (2013).

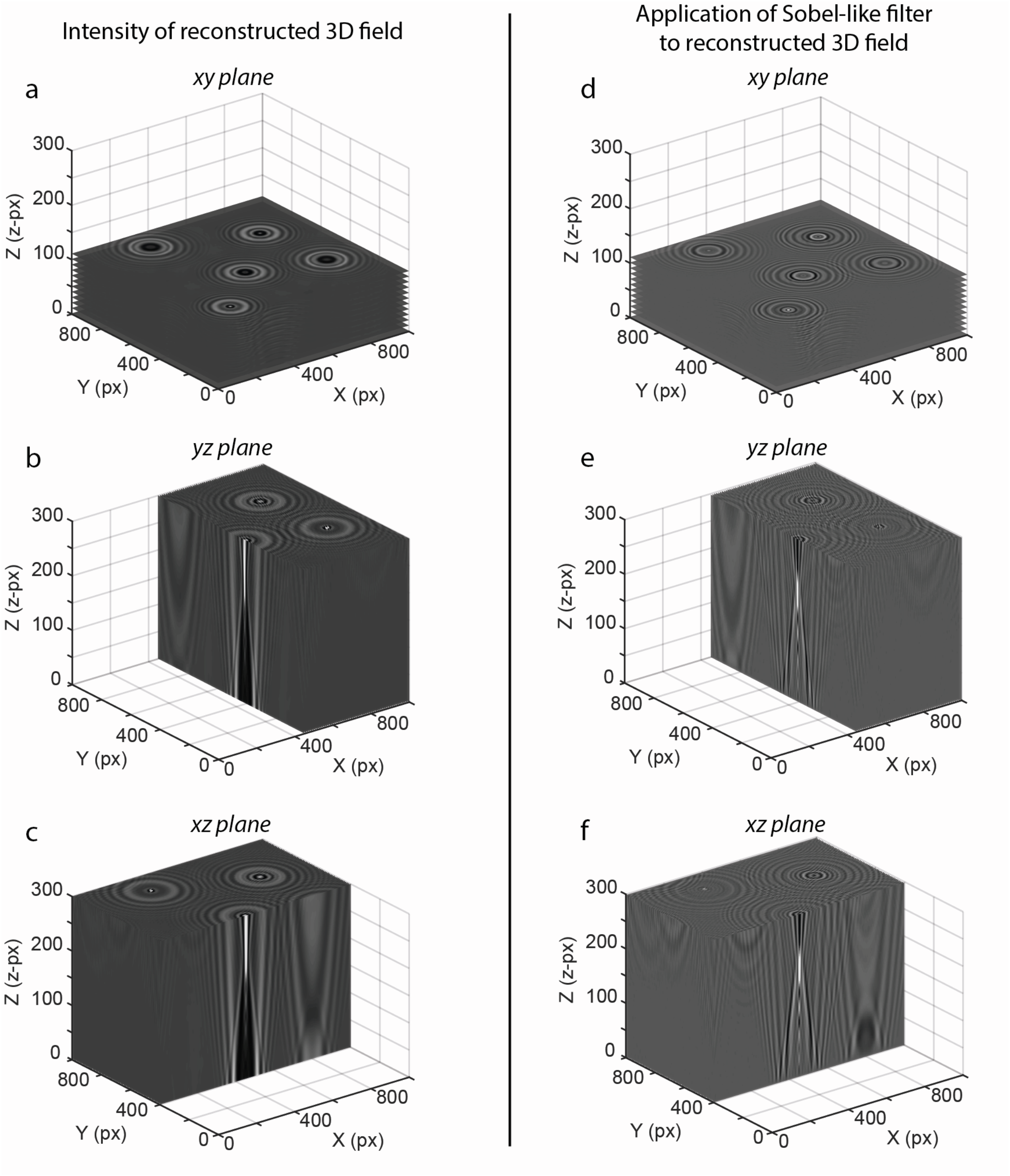

Supplementary Figure 1. Holographic reconstruction and the effect of applying a 3D Sobel-like filter. The intensity of the back-propagated 3D electromagnetic field from the hologram shown in Fig. 1c is displayed in (a), (b) and (c) with cutaways along different planes. (d), (e) and (f) show the same cutaways after the Sobel-like filter has been applied to the data, resulting in the gradient of the field along z. Features in the 3D field, such as the bright spots corresponding to the positions of microbeads, are now easier to detect. Supplementary videos 1 to 6 show animations scrolling through these reconstructed volumes. Pixels along x and y are 0.065 µm; each pixel value along z corresponds to 0.1 µm.

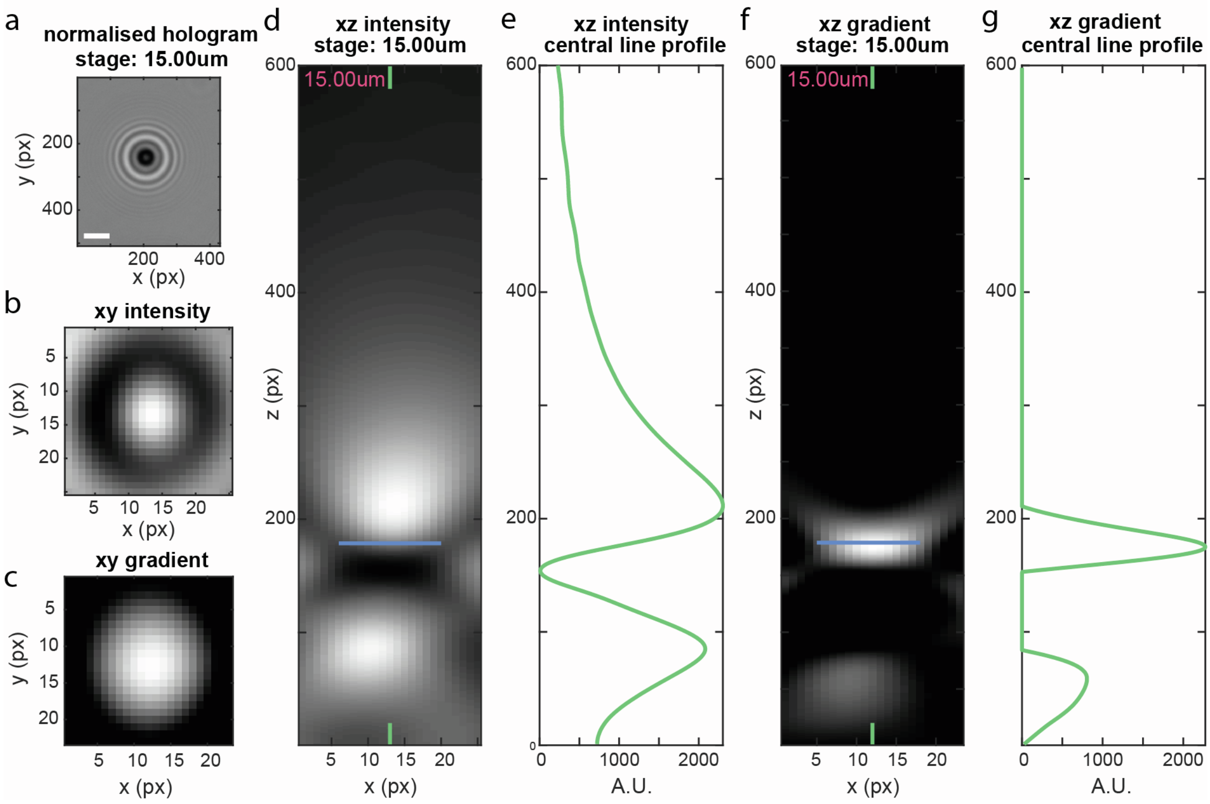

Supplementary Figure 2. Application of 3D Sobel-like filter to holographic reconstruction. (a) Recorded normalised hologram, of a 2.8 µm-diameter Dynabead. The stage is positioned 15 µm below the focal plane. The scale bar is 5 µm. (b) Zoom-in of the intensity of the holographic reconstruction *at the z-value of initial object detection* (blue horizontal lines in (d) and (f)). (c) Same plane as in (b) but after the Sobel-like gradient filter has been applied. (d),(f) xz-planes through the centre of the microsphere from the focal plane (1) to the extent of the holographic reconstruction (600). (d) Intensity of the reconstructed field; (f) the field after the Sobel-like gradient filter has been applied. (e), (g) Vertical line profiles through the centres of (d) and (f), respectively. The taller peak of the trace in (g) is used as a proxy for position of the microbead along z. The value of this peak (blue line in (d) and (f)) is the initial guess fed into the 3D parabolic masking algorithm for more precise localisation. Supplementary video 7 shows an animation of these data scrolling through all reconstructed planes to a distance of 35 µm from the focus. Pixels along x and y are 0.065 µm wide; each pixel value along z corresponds to 0.1 µm. A.U. = arbitrary units.

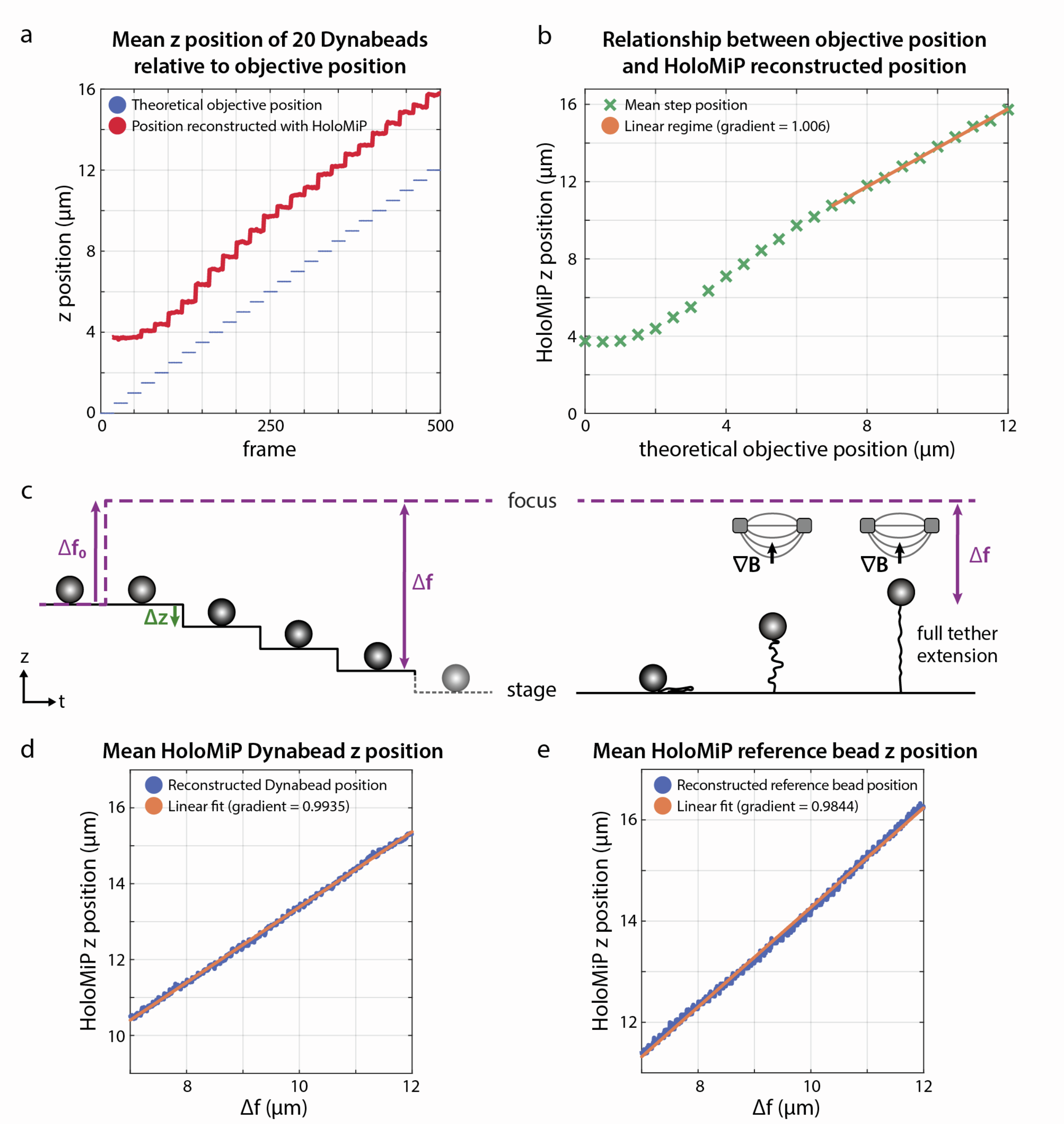

Supplementary Figure 3. Optical correction for 3D imaging with 40x objective. (a) After focusing on the floor of a sample of immobilised microbeads, the objective lens is moved in discrete steps (blue). The 3D positions of beads are recovered using HoloMiP (red). (b) We observe a linear relationship between objective position and z position determined by HoloMiP when the objective is positioned between 7 and 12 μm from the sample. (c) An experiment to validate this result begins by moving the objective focus a distance Δf_0 from the sample chamber floor. The stage is then moved away from the focus in discrete steps Δz. This simulates the behaviour of a Dynabead in a magnetic tweezers experiment (right). We find the linear relationship between sample-to-focus distance (Δf) and z position recovered using HoloMiP holds for the range of 7 to 12 μm for both Dynabeads (d) and silica reference beads (e).

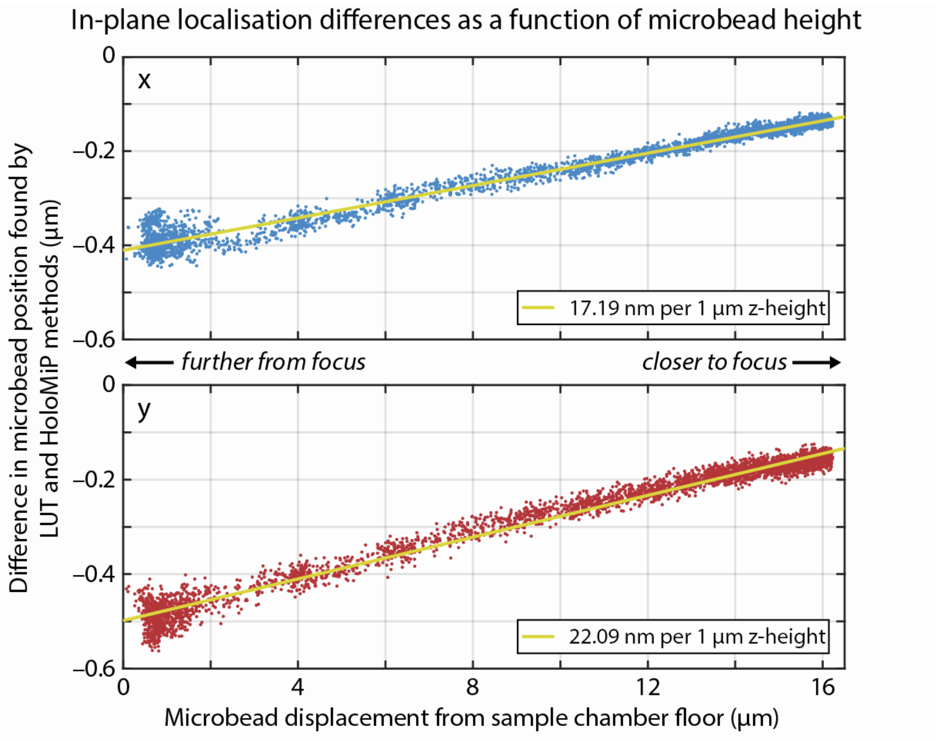

Supplementary Figure 4. The 3D position of a 2.8 µm-diameter Dynabead tethered to a 16.3 µm DNA tether in response to an increasing magnetic field was determined by both the look-up table cross-correlation method (LUT) and HoloMiP. The difference between these two techniques shows an offset along both the x (top) and y (bottom) axes, which correlates to a linear function (yellow line). As stated in the text, we conclude this offset is a function of the way in which the LUT technique averages a 2D diffraction pattern to produce a 1D radial profile, thus imposing an artificial radial symmetry on the ring patterns and rendering the technique insensitive to minute differences in angle between incident light and the normal to the imaging plane. The holographic reconstruction in HoloMiP does not involve radial averaging. We determined an angle between optical axis and normal to the imaging plane of around 1.1°.

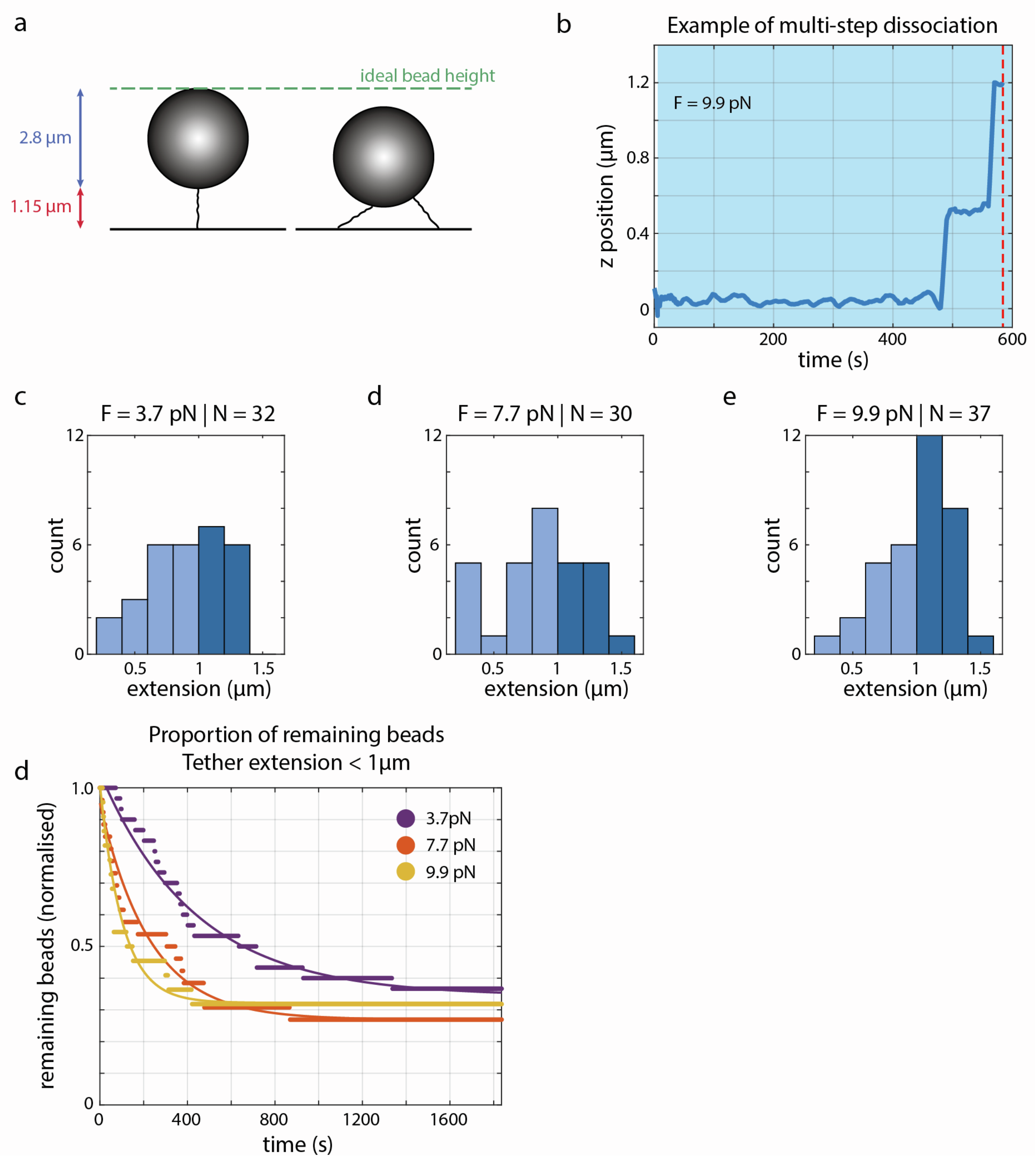

Supplementary Figure 5. Evidence of multiple tether attachments in MT experiments. (a) Scale diagram showing Dynabeads with multiple tether attachments will not extend to the ideal bead height in magnetic tweezer experiments. (b) Example z position trace of a Dynabead exhibiting multi-step dissociation in CR2–anti-CR2 force-mediated unbinding experiments. The blue shading indicates the applied force; the red dashed line indicates when the microbead fully dissociates. (c, d, e) Histograms for each applied force showing the extensions that tethered microbeads reached before dissociation. We selected microbeads that reached 1 µm extension (dark shading) for the analysis shown in Fig. 4. The remaining beads were analysed in (d) and (e). Beads exhibiting multi-step behaviour were excluded. (d) Proportion of microbeads with a tether extension < 1 µm in the

CR2–anti-CR2 dataset remaining attached through time. Single exponentials could only be fitted with a high plateau term to account for the large proportion of microbeads that did not dissociate by the end of the recording (F = 3.7 pN, N = 19 with 11 remaining at end of recording, R^2 = 0.989; F = 7.7 pN, N = 19 with 7 remaining at end, R^2 = 0.985; F = 9.9 pN, N = 15 with 7 remaining at end, R^2 = 0.985).

Supplementary video 1. xy planes of 3D intensity field reconstruction (related to Supplementary Fig. 1a).

Supplementary video 2. yz planes of 3D intensity field reconstruction (related to Supplementary Fig. 1b).

Supplementary video 3. xz planes of 3D intensity field reconstruction (related to Supplementary Fig. 1c).

Supplementary video 4. xy planes of 3D gradient field reconstruction (related to Supplementary Fig. 1d).

Supplementary video 5. yz planes of 3D gradient field reconstruction (related to Supplementary Fig. 1e).

Supplementary video 6. xz planes of 3D gradient field reconstruction (related to Supplementary Fig. 1f).

Supplementary video 7. Animation showing effect of Sobel-like gradient filter (related to Supplementary Fig. 2).
